# An *in vitro* assay to measure antibody-mediated inhibition of *P*. *berghei* sporozoite invasion against *P*. *falciparum* antigens

**DOI:** 10.1038/s41598-017-17274-5

**Published:** 2017-12-05

**Authors:** Ana Rodríguez-Galán, Ahmed M. Salman, Georgina Bowyer, Katharine A. Collins, Rhea J. Longley, Florian Brod, Marta Ulaszewska, Katie J. Ewer, Chris J. Janse, Shahid M. Khan, Julius C. Hafalla, Adrian V. S. Hill, Alexandra J. Spencer

**Affiliations:** 1 The Jenner Institute, University of Oxford, Oxford, United Kingdom; 2 Department of Immunology and Infection, Faculty of Infectious and Tropical Diseases, London School of Hygiene and Tropical Medicine, London, United Kingdom; 3 Department of Parasitology, Leiden University Medical Center, Leiden, The Netherlands; 4 Present Address: Walter and Eliza Hall Institute of Medical Research, Parkville, Victoria, Australia

## Abstract

A large research effort is currently underway to find an effective and affordable malaria vaccine. Tools that enable the rapid evaluation of protective immune responses are essential to vaccine development as they can provide selection criteria to rank order vaccine candidates. In this study we have revisited the Inhibition of Sporozoite Invasion (ISI) assay to assess the ability of antibodies to inhibit sporozoite infection of hepatocytes. By using GFP expressing sporozoites of the rodent parasite *P*. *berghei* we are able to robustly quantify parasite infection of hepatocyte cell lines by flow cytometry. In conjunction with recently produced transgenic *P*. *berghei* parasites that express *P*. *falciparum* sporozoite antigens, we have been able to use this assay to measure antibody mediated inhibition of sporozoite invasion against one of the lead malaria antigens *P*. *falciparum* CSP. By combining chimeric rodent parasites expressing *P*. *falciparum* antigens and a flow cytometric readout of infection, we are able to robustly assess vaccine-induced antibodies, from mice, rhesus macaques and human clinical trials, for their functional ability to block sporozoite invasion of hepatocytes.

## Introduction

Although there has been an estimated 40% reduction in the incidence of *P*. *falciparum* malaria infections over the last 15 years, owing to the wider deployment of multiple malaria intervention strategies^1^, it continues to cause significant mortality and morbidity. The WHO estimates that there were half a million malaria associated deaths in 2014, with the majority in sub-Saharan Africa, in children under the age of 5^2^. Reports of increasing anti-malarial drug resistance highlight the vital importance of a malaria vaccine.

A malaria infection in humans starts with the bite of an infected mosquito which injects *P*. *falciparum* sporozoites into the skin as it takes a blood meal. Sporozoites then migrate to the liver where they infect hepatocytes and undergo asexual replication, subsequently leading to the formation and release of merozoites into the blood stream approximately 6–7 days later, which infect and re-infect red blood cells (RBCs).

Significant clinical advances have been made with two pre-erythrocytic stage recombinant vaccines against two different *P*. *falciparum* sporozoite stage proteins. RTS,S is an antibody inducing vaccine that targets the circumsporozoite protein (CSP)^3^. The other vaccine is heterologous viral vector vaccination with simian Adenovirus serotype 63 (ChAd63) followed by modified vaccinia Ankara (MVA), both expressing a multiple epitope (ME) string fused to thrombospondin related anonymous protein (TRAP) and this primarily induces effector CD8^+^ T cells against TRAP to kill infected hepatocytes, although anti-*Pf*TRAP antibodies are also induced^4^. While both vaccines have shown some degree of efficacy in African clinical trials^5,6^, which was marginally enhanced (75% to 82.4% sterile efficacy) when vaccines were combined in malaria naïve individuals^7^, further vaccine development and regimen optimisation will be required to achieve long-term efficacy in the vaccine target population.

Antibodies generated against *Pf*CSP have been shown to block sporozoite invasion of hepatocytes^8^ and protection afforded by vaccination with RTS,S, a virus-like particle (VLP) expressing the repeat region and C-terminal domain of CSP, correlates primarily with the level of anti-*Pf*CSP antibodies^9^. In order to identify novel candidate vaccine antigen targets and/or different vaccine strategies, sensitive assays are required to measure the effect of antibodies on sporozoite survival and hepatocyte invasion. However, unlike the growth inhibition assay (GIA) that can be used to functionally assess vaccines against the blood-stage of malaria, there are currently no standardized rapid screening assays to measure the capacity of antibodies to inhibit invasion of hepatocytes by sporozoites. Such assays are lacking, in part due to limited access to *P*. *falciparum* sporozoites, but also the limited number (and availability) of *P*. *falciparum* infection-permissive hepatocyte cell lines and the lack of a medium to high throughput method to measure the number of infected hepatocytes.

The first study to measure the capacity of antibodies to prevent sporozoite invasion utilised a human embryonic lung cell line and staining for intra-cellular parasites by either Giemsa or immunofluorescence^10^. Following on from this, a number of different groups used hepatocyte cell lines to determine the capacity of antibodies against *P*. *falciparum* or *P*. *vivax* antigens to inhibit sporozoite infection by histological analysis^11–13^. While labelled antibodies^13–15^ and RT-PCR^16^ have been previously used as a quantitative readout of sporozoite invasion, these methods are labour-intensive and costly to perform.

With the advances in transgenic parasite technology it is now possible to generate chimeric *P*. *berghei* parasites that express *P*. *falciparum* antigens, and these parasite lines are capable of infecting mice and hepatocyte cell lines^17^. In addition, a variety of *P*. *berghei* parasites expressing fluorescent markers that can be detected by flow cytometry are now available. Using a transgenic *P*. *berghei* line that expresses GFP in sporozoites and during liver-stage infection, we recently developed an *in vitro* T cell killing assay and were able to demonstrate killing of malaria infected hepatocytes by effector CD8^+^ T cells (obtained from viral vector vaccinated mice) with a flow cytometric readout^18^.

In this current study, we developed a new inhibition of sporozoite invasion (ISI) assay, based on a flow cytometric readout, to assess the ability of antibodies induced by immunisation, for their ability to inhibit sporozoite infection of hepatocytes. Using chimeric *P*. *berghei* parasites expressing the lead *P*. *falciparum* vaccine antigens, CSP or TRAP, we have been able to use this assay to test the function of antibodies induced in mice, primate and human vaccination studies. By combining the advances of chimeric parasite technology and flow cytometry we have established a medium to high-throughput functional assay, which can be used as an *in vitro* readout of immunisation efficacy of antibodies targeting sporozoite antigens and has scope to test many additional *P*. *falciparum* antigens.

## Results

### Optimising hepatocyte and sporozoite cell numbers for the ISI assay

For the studies described below two transgenic *P*. *berghei* parasite lines were used; *Pb*GFP which expresses GFP or *Pb*GFP-Luc which expresses a fusion protein of GFP and luciferase both under control of the constitutive *Pbeef1α* promoter. The chimeric *P*. *berghei* parasites expressing *P*. *falciparum* antigens (see below) also express the GFP-luciferase fusion protein under control of the *eef1α* promoter.

**Figure 1:**
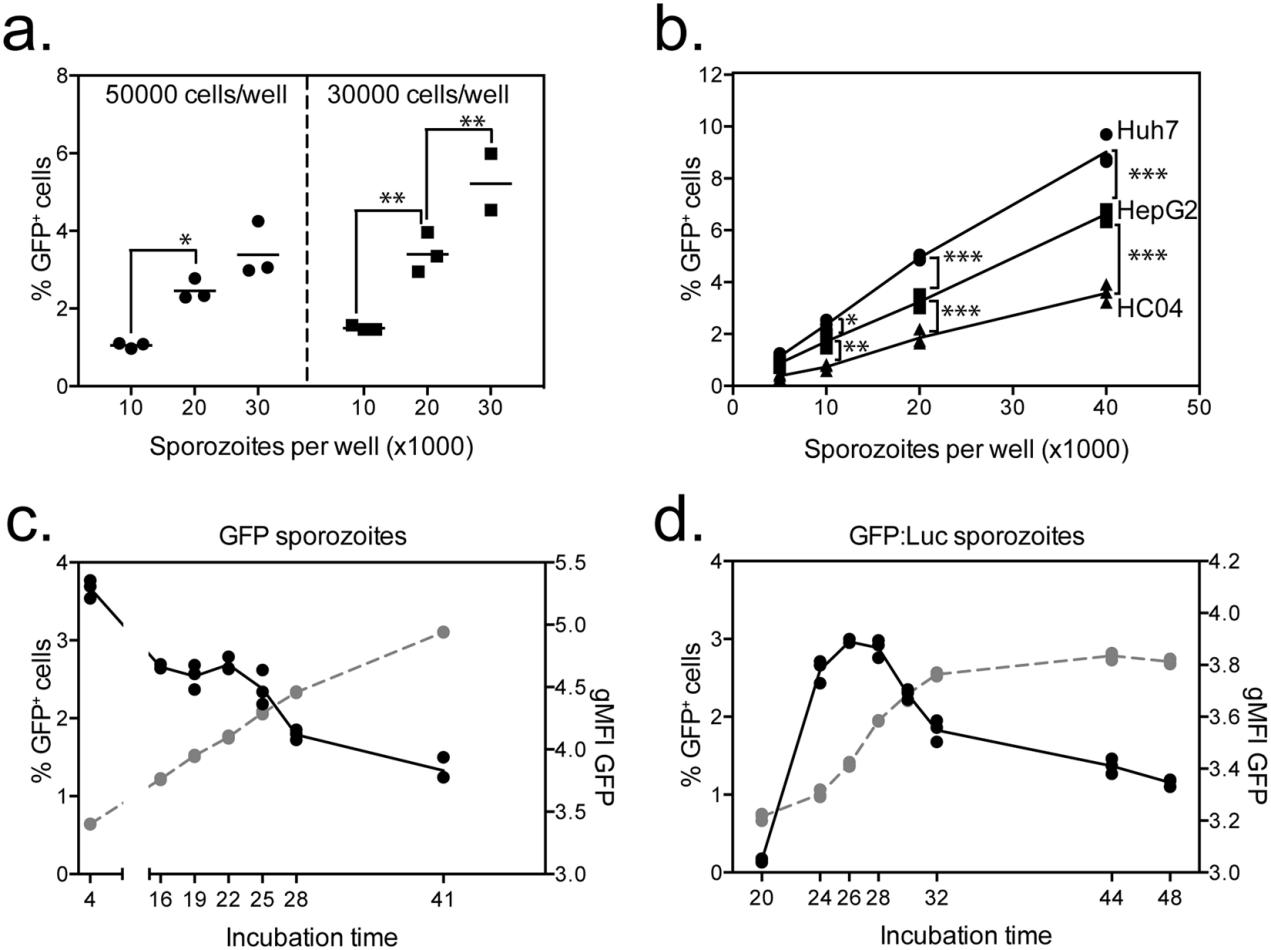
Optimisation of culture conditions for detection of infection *P*. *berghei* by flow cytometry. (**a**) 50000 or 30000 HepG2 cells were plated per well and infected with 10000, 20000 or 30000 *Pb*GFP sporozoites and harvested the following day to measure the percentage of infected cells by flow cytometry. (**b**) 30000 Huh7, HepG2 or HC04 cells were seeded per well and subsequently infected with increasing numbers of *PbGFP* sporozoites. Cells were harvested the following day and the percentage of infected cells determined by flow cytometry. (**c**) 30000 Huh7 cells per well were infected with 15000 *PbGFP* sporozoites and the percentage of infected cells was measured between 4 and 41 hours after incubation. The graph represents the percentage of GFP^+^ cells (left axis) (black line) or log Mean Fluorescence Intensity (MFI) of GFP from GFP^+^ cells (right axis) (grey line) against the time after infection. (**d**) 30000 Huh7 cells per well were infected with 20000 *Pb*GFP-Luc sporozoites and the percentage of infected cells was measured between 20 and 48 hours after incubation. The graph represents the percentage of infected cells (left axis) (black line) or log Mean Fluorescence Intensity (MFI) of GFP in GFP^+^ cells (right axis) (grey line) against the time after infection.

As different hepatocyte cell lines have different permissiveness to *P*. *berghei* sporozoite infection with varying parasite growth rates, initial experiments were performed to identify the optimal cell-culture conditions for the ISI assay to reliably detect differences in sporozoite infectivity by flow cytometry. In an initial experiment, HepG2 cells were seeded at two cell concentrations (30000 or 50000 cells per well) and infected with different number of *Pb*GFP sporozoites (30000, 20000, 10000). Plates were incubated overnight prior to cell harvesting and acquisition of samples on the flow cytometer. Plating 30000 HepG2 per well led to higher percentages of infected cells compared to wells of 50000 cells (Fig. 1a) and this was consistent for any number of sporozoites added. Importantly, the same percentage of infected cells was achieved when the ratios of sporozoites to hepatocytes were compared; 30000 sporozoites and 50000 cells (1:1.6 sporozoite:cell ratio) gave a similar level of infectivity as 30000 cells infected with 20000 sporozoites (1:1.5 sporozoite:cell ratio). The ability of different numbers of *Pb*GFP sporozoites to infect Huh7 and HC04 cells was also compared. Addition of higher numbers of sporozoites led to an increased percentage of infected cells (Fig. 1b) for all three cell lines tested. Huh7 cells showed the highest permissiveness for *P*. *berghei* as a higher percentage of infected Huh7 cells was observed, compared to HepG2 or HC04 cells, regardless of the number of sporozoites added per well (Fig. 1b). As each cell line has preferential cell growth medium that could affect sporozoite infectivity, we compared different combinations of cell lines and media to identify the optimal culture conditions. Although culturing cells in different media had only a small effect on the percentage of infected cells, the highest infectivity was observed with complete RPMI medium (Figure S1a) and therefore RPMI was used in all further experiments.

As the readout of sporozoite infectivity relies on expression of GFP from intracellular parasites and detection of this GFP signal by flow cytometry, it was important to identify the optimal time after the addition of sporozoites to harvest cells and acquire samples on the flow cytometer. Cells infected with *Pb*GFP sporozoites could be detected by flow cytometry as early as 4 hours post infection (hpi) (Fig. 1c) which had dropped by 16 hours post infection, reflective of high number of transversal or aborted invasion events which can be detected by flow cytometry at early hours post infection^19^. The percentage of infected cells continued to slowly decline over time from 16 hours post-infection, most likely due to replication of non-infected Huh7 cells in the wells (Bliss *et al*. in preparation). Although the percentage of infected cells decreased with time, the mean fluorescent intensity (MFI) of the GFP signal increased, consistent with an increase in exoerythrocytic parasite forms within the hepatocyte^20^. In contrast to *Pb*GFP infected hepatocytes, a GFP signal was not detected from hepatocytes infected with *Pb*GFP-Luc sporozoites before 20 hpi (Fig. 1d), with the percentage of GFP-positive cells increasing after 20 hpi with a peak at 26 hpi. The reduced GFP signal intensity of *Pb*GFP-Luc infected hepatocytes compared to *Pb*GFP infected hepatocytes is due to reduced GFP-fluorescence when GFP is fused to luciferase^21^.

GFP MFI signal also increased over time in *Pb*GFP-Luc infected hepatocytes, consistent with *Pb*GFP parasites, indicating an increased growth of the liver stage parasite within the hepatocytes. Based on these results, 24–28 hours after infection was chosen as the optimal time to quantify *Pb*GFP-Luc and *Pb*GFP infected cells by flow cytometry.

### An anti-*P*. *berghei* CSP monoclonal antibody inhibits sporozoite invasion of hepatocytes in a dose dependent manner in the ISI assay

**Figure 2:**
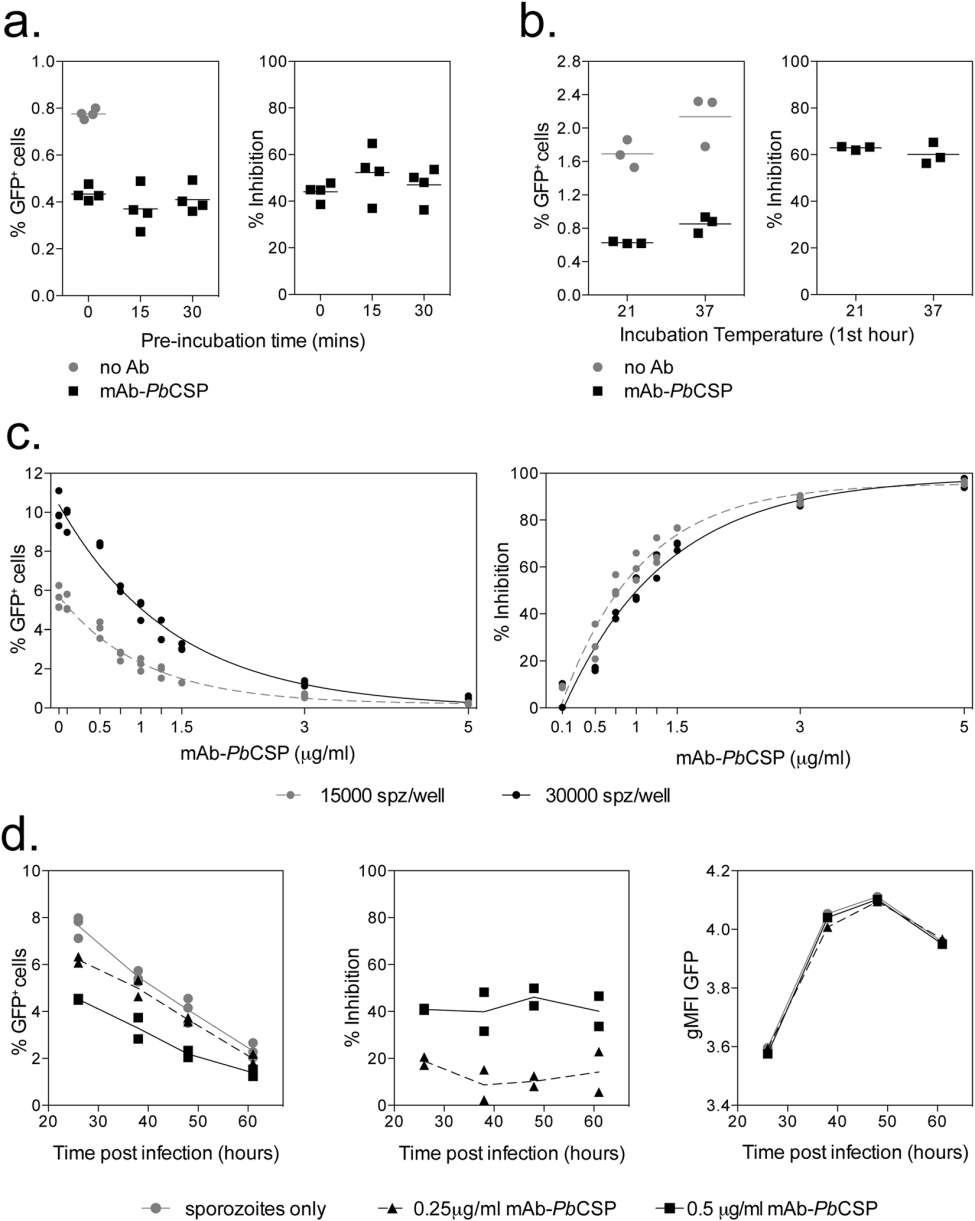
Optimising the detection of antibody mediated inhibition of sporozoite invasion. (**a**) 10000 *PbGFP* sporozoites were incubated with 1 μg/mL mAb-*Pb*CSP (3D11) on ice for 0, 15 or 30 minutes prior to addition to 30000 Huh7 cells. Cells were harvested the following day and the percentage of GFP^+^ cells determined by flow cytometry. The graphs represent the percentage of GFP^+^ cells (left) or percentage of inhibition (right) when compared to the sporozoite only control well. (**b**) 20000 *PbGFP* sporozoites together with 1 μg/ml mAb-*Pb*CSP were added to 30000 Huh7 cells and incubated at 21 ^o^C or 37 ^o^C for 1 hour, prior to overnight incubation at 37 ^o^C. Cells were harvested the following day and the percentage of GFP^+^ cells determined by flow cytometry. Graphs represent the percentage of GFP^+^ cells (left) or percentage of inhibition (right) when mAb-*Pb*CSP wells were compared to sporozoite only wells incubated under the same conditions. (**c**) 30000 (black) or 15000 (grey) sporozoites (*Pb*GFP-Luc) were added to 30000 Huh7 cells in the presence of a range of mAb-*Pb*CSP concentrations (0–5 μg/mL) and percentage of infection measured by flow cytometry the following day. Graphs represent the percentage of GFP^+^ (left) or percentage of inhibition (right) when addition of mAb-*Pb*CSP was compared to sporozoite only control wells. Data was analysed with a two-way ANOVA which showed a significant effect of antibody concentration but no significant effect of sporozoite number. (**d**) 15000 sporozoites (*Pb*GFP-Luc) were added to 30000 Huh7 cells per well in the presence of mAb-*Pb*CSP (0.25 or 0.5 μg/mL) and harvested at different times after infection. Graphs represent the percentage of GFP^+^ cells over time (left), percentage of inhibition (middle) or log GFP MFI of infected cells over time (right). The percentage of inhibition data was analysed with a two-way ANOVA to test for the effect of antibody concentration over time, no significant effect of time was observed. Log (MFI GFP^+^) data was analysed with a two-way ANOVA and post-hoc Dunn’s multiple comparisons test, but no difference between groups at each timepoint was observed.

To optimise the culture and incubation periods required to detect antibody mediated inhibition of sporozoite invasion, initial experiments were performed with a monoclonal antibody (mAb) against *P*. *berghei* CSP (3D11, mAb-*Pb*CSP). In the first experiment we wished to determine whether pre-incubation of sporozoites with mAb-*Pb*CSP for different periods of time would have an effect on sporozoite invasion. Addition of 1 μg/ml of mAb-*Pb*CSP antibody was shown to reduce the percentage of infected cells; however no significant differences were observed in infectivity when sporozoites were directly added, or pre-incubated with mAb-*Pb*CSP for 15 or 30 minutes prior to addition of the sporozoite-mAb mixture to the hepatocytes, in terms of the percentage of infected cells (Fig. 2a left) or when calculated as a percentage of inhibition compared to the sporozoite only wells (Fig. 2a right).

Infectivity of sporozoites is very sensitive to temperature changes. Incubation for 30 minutes at 37 ^o^C before addition to hepatocytes, has been shown to reduce infectivity by 80%^22^, and an increase in temperature from 20 ^o^C to 37 ^o^C in the presence of serum is sufficient to induce the transformation of sporozoites into exo-erythrocytic forms in the absence of hepatocytes^23^. We therefore determined whether maintaining cells at 19–21 ^o^C, the optimal temperature for *P*. *berghei* sporozoite development in mosquitoes^24^, improved sporozoite infectivity or altered the ability of mAb to block the invasion of sporozoites. Importantly we found that the incubation temperature for the first hour of culture, be it 21 ^o^C or 37 °C, did not appear to have an effect on either the infectivity of the cells (Fig. 2b left), or the ability of mAb-*Pb*CSP to block sporozoite invasion (Fig. 2b right). Therefore in all further experiments serum/mAb and sporozoite were added directly to the wells, prior to centrifugation and incubation at 37 ^o^C.

To determine whether inhibition of sporozoite invasion was dependent on the concentration of the mAb used, different mAb-*Pb*CSP concentrations, from 0.1 to 5 μg/mL, were mixed with either 15000 or 30000 *Pb*GFP-Luc sporozoites. mAb-*Pb*CSP was found to inhibit the ability of sporozoites to infect Huh7 hepatocytes in a concentration-dependent manner, with between 50–60% blocking at 1 μg/mL, and 96% inhibition at 5 μg/mL (Fig. 2c left), while at 0.1 μg/mL only 3% inhibition was observed. The percentage of infected cells observed was doubled when infected with 30000 compared to 15000 sporozoites (Fig. 2c left), and similar drug inhibition curves were obtained with both 15000 and 30000 sporozoites infections. Halving the sporozoite numbers did not alter the dose response curve, which indicated that fewer sporozoites per well (15000) can be used without decreasing assay sensitivity. This would also suggest that fluctuations in sporozoite infectivity (or the error associated with the sporozoite counts) should not impact the ISI readout, as long as the infectivity data is analyzed in each experiment with reference to the sporozoite infectivity in control wells. Importantly, similar inhibition curves were observed when either 15000 or 30000 sporozoites were added, although the percentages of inhibition were slightly higher when 15000 sporozoites were added (albeit not statistically significant), suggesting a potential limiting effect of antibody when more sporozoites are added per well.

To determine whether mAb-*Pb*CSP was only blocking sporozoite invasion of hepatocytes or if it was also affecting parasite growth within the hepatocytes, *Pb*GFP-Luc sporozoites were added in the presence of mAb-*Pb*CSP at 0.25 and 0.5 μg/mL and cells harvested at different time periods to measure the number of infected cells and the level of the GFP signal. Although the percentage of infected cells decreased over time (Fig. 2d left), consistent with initial experiments, the percentage of sporozoite inhibition observed by the addition of 0.25 or 0.5 μg/ml of mAb-*Pb*CSP remained consistent over time (Fig. 2d middle). In addition, the level of GFP signal was not significantly different between the groups at any time (Fig. 2d right), consistent with the monoclonal antibody only blocking the ability of sporozoites to invade the hepatocytes and not affecting their subsequent growth within hepatocytes. While the time of readout is important in terms of the ability to detect a GFP signal by flow cytometry (Fig. 1), the data demonstrate the robustness of the assay, as the percentage of inhibition remains constant regardless of the time that cells are harvested. However due to inter experiment variability in sporozoite infectivity which can lead to changes in the percentage of inhibition observed (Fig. 2a,b,d), comparisons between groups should only be performed within a single experiment.

In addition to having established the optimal conditions to determine antigen specific inhibition, we tested the effect of serum concentration and heat inactivation on inhibition of sporozoite invasion. In these experiments we used serum collected from a naïve mouse. Increasing the concentration of serum resulted in a decreased number of infected cells (Figure S1b left) and therefore increased percentage of inhibition (Figure S1b middle). Heat inactivation appeared to increase inhibition of sporozoite invasion (albeit not statistically significantly), although it is unlikely that this is antibody mediated inhibition as heat inactivation of mAb-*Pb*CSP abolished its sporozoite inhibitory effect (Figure S1b right). The increased inhibition of sporozoite invasion observed with increasing serum concentrations is most likely due to the role of serum, and culture temperature, in inducing transformation of sporozoites into exo-erythrocytic forms^23^, thereby reducing sporozoite infectivity. Given the increased inhibition of sporozoite invasion, or decreased sporozoite infectivity, observed with increasing serum concentrations and the variability that can occur between experiments, it is important to include controls with pre-vaccination or naïve serum in all experiments, to ensure inhibition is antigen specific.

### The ISI assay using antibodies induced by vaccination with vectored vaccines expressing *Pf*CSP

**Figure 3:**
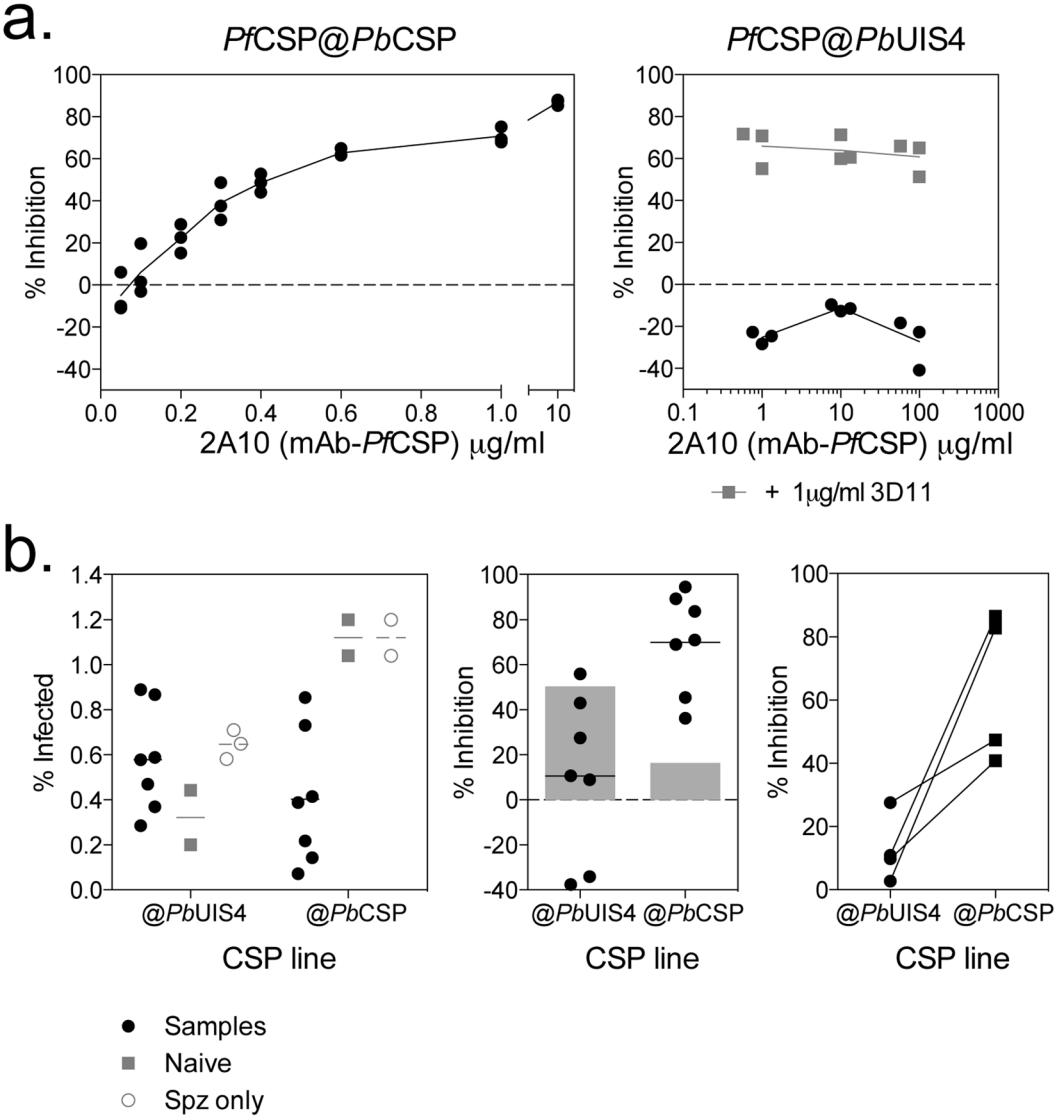
Inhibition of *Pf*CSP@*Pb*CSP and *Pf*CSP@*Pb*UIS4 parasites with monoclonal antibodies. (**a**) 15000 sporozoites *Pf*CSP replacement (*Pf*CSP@*Pb*CSP) (left) or *Pf*CSP addition (*Pf*CSP@*Pb*UIS4) (right) were added to 30000 Huh7 cells with increasing concentrations of mAb-*Pf*CSP (2A10), with the addition of mAb-*Pb*CSP (3D11) at 1 μg/mL for *Pf*CSP addition parasites (grey squares). Cells were harvested 24 to 28 hours later and the percentage of infected cells determined by flow cytometry. Graphs represent the percentage of inhibition relative to sporozoite only control wells, calculated for each replicate at each dose. (**b**) 20000 Huh7 cells per well were cultured overnight prior to the addition of either 10000 *Pf*CSP@*Pb*CSP or *Pf*CSP@*Pb*UIS4 sporozoites together with serum samples from ChAd63-MVA vaccinated mice (5% final concentration of serum) (in a separate experiment from Fig. 4). Cells were harvested 24 to 28 hours later and the percentage of infected cells determined by flow cytometry. Graphs represent the frequency of GFP + cells (left), percentage of inhibition (middle) against each parasite strain or when comparing the same serum sample tested with either cell line (right). Grey bars denote the percentage of inhibition observed in the assay with naïve serum samples.

Having optimized the ISI assay, we wished to use it to test the inhibitory effect of antibodies against CSP of the human parasite *P*. *falciparum*. For these experiments we used a chimeric *P*. *berghei* line that has the endogenous *csp* gene replaced with the *P*. *falciparum csp* gene (called *Pf*CSP@*Pb*CSP). Sporozoites of this chimeric line expresses only *P*. *falciparum* CSP (*Pf*CSP) and it produces normal numbers of sporozoites that have similar infectivity to HepG2 as wild type *P*. *berghei* sporozoites (Triller G, Scally SW *et al*., submitted). We first tested inhibition of invasion of *Pf*CSP@*Pb*CSP sporozoites by the anti-*Pf*CSP mAb 2A10 and a clear dose effect was observed when increasing concentrations of 2A10 were used (Fig. 3a left). We also tested the ability of anti-*Pf*CSP 2A10 monoclonal antibody to inhibit *P*. *berghei* parasites expressing the *Pf*CSP gene when expressed as an additional gene under the control of the *Pb*UIS4 promoter (*Pf*CSP@*Pb*UIS4). Despite the ability of anti-*Pb*CSP 3D11 monoclonal antibody to inhibit invasion (Figure S2b), to our surprise, increasing concentrations of the *Pf*CSP specific monoclonal antibody 2A10 were unable to inhibit invasion when parasites expressed both copies of the CSP protein, and it was only in the presence of *Pb*CSP specific mAb 3D11 when inhibition was observed (Fig. 3a right). Interestingly, 2A10 monoclonal antibody is able to bind to the sporozoite surface of both *Pf*CSP@*Pb*CSP and *Pf*CSP@*Pb*UIS4 (Figure S2a), even though inhibition against *Pf*CSP@*Pb*CSP in the ISI assay is not observed (Figure S2b). When serum samples from *Pf*CSP vaccinated mice were used to compare inhibition observed with each parasite line, although some degree of inhibition with both parasites lines was observed, significant inhibition above the naïve samples was more apparent with the replacement *Pf*CSP@*Pb*CSP (Fig. 3b). Given the critical role of CSP in cell invasion, we hypothesized that in conditions of high anti-*Pf*CSP antibodies, parasites may revert to invasion of hepatocytes through *Pb*CSP and that this may be antibody dose dependant.

**Figure 4:**
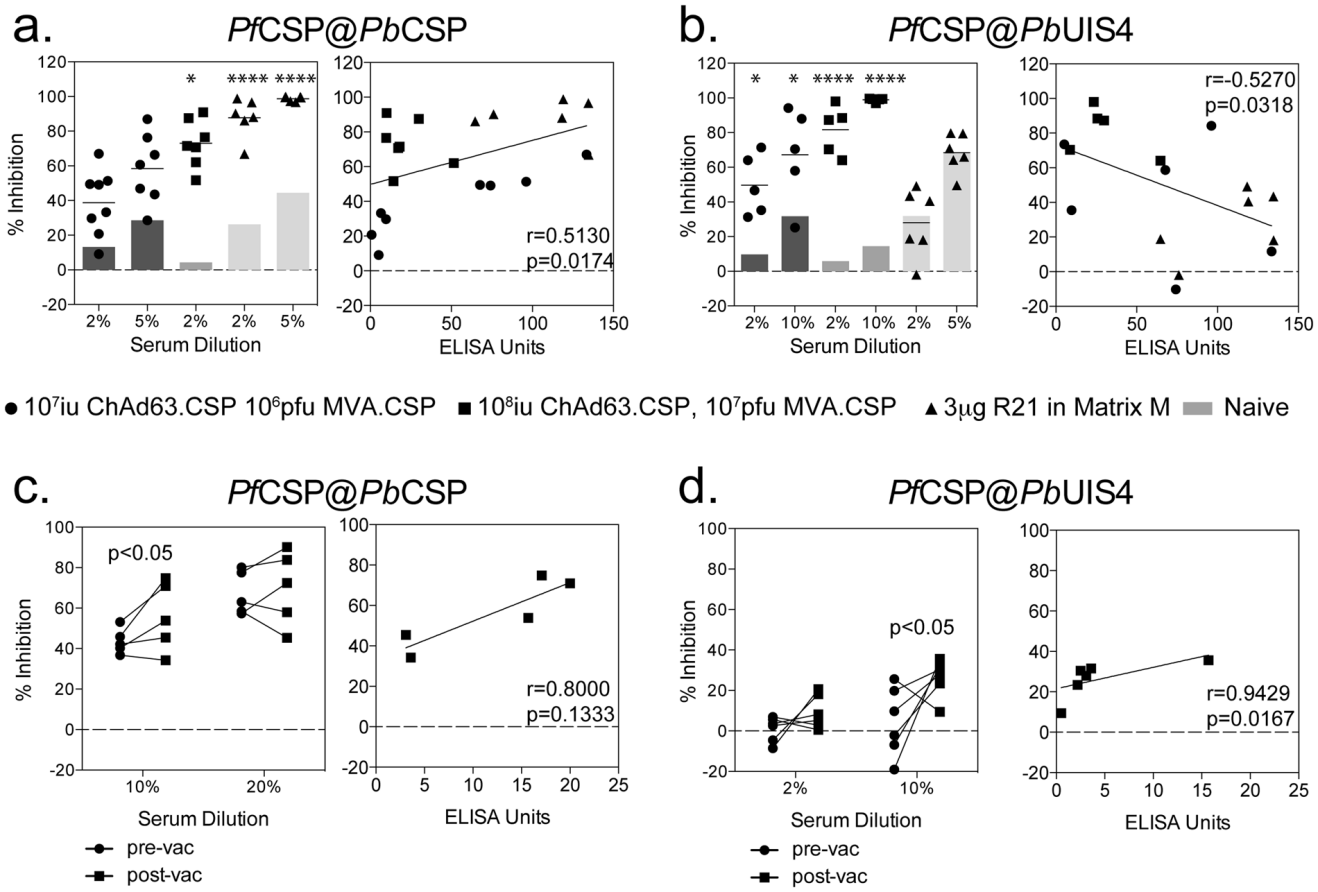
Inhibition of *Pf*CSP@*Pb*CSP and *Pf*CSP@*Pb*UIS4 parasites with *Pf*CSP vaccinated serum samples. (**a**) In 3 separate experiments, serum from BALB/c mice vaccinated with 10^7^ iu ChAd63.*Pf*CSP 10^6^ MVA.*Pf*CSP (filled circle), 10^8^ iu ChAd63.*Pf*CSP 10^7^ pfu MVA.*Pf*CSP (closed squares) or 3 μg R21 in Matrix M (filled triangles) were tested for the ability to inhibit the invasion of Huh7 cells with *Pf*CSP@*Pb*CSP sporozoites. In each experiment, the grey bars denote the level of inhibition observed with naïve serum samples. Data in each graph was analysed with a two-way ANOVA and post-hoc Sidaks multiple comparison, asterisk denote the level of significance comparing *Pf*CSP samples against naïve serum samples, *p < 0.05 ****p < 0.0001. The percentage of inhibition observed with 2% serum concentration was compared to antibody titres measured by ELISA (right) and analysed with a two-tailed Spearmans correlation. (**b**) In 3 separate experiments, serum from BALB/c mice vaccinated with 10^7^ iu ChAd63.CSP 10^6^ MVA.CSP (filled circle), 10^8^ iu ChAd63.CSP 10^7^ pfu MVA.CSP (closed squares) or 3 μg R21 in Matrix M (filled triangles) were tested for the ability to inhibit the invasion of Huh7 cells with *Pf*CSP@*Pb*UIS4 sporozoites. In each experiment, the grey bars denote the level of inhibition observed with naïve serum samples. Data in each graph was analysed with a two-way ANOVA and post-hoc Sidaks multiple comparison, asterisk denote the level of significance comparing *Pf*CSP samples against naïve serum samples, *p < 0.05 ****p < 0.0001. The percentage of inhibition observed with 2% serum dilutions was compared to antibody titres measured by ELISA (right) and analysed with a two-tailed Spearmans correlation (p = 0.0318). (**c**) Serum samples from human volunteers vaccinated with a ChAd63.*Pf*CSP followed by MVA.*Pf*CSP were tested for the ability to inhibit the invasion of Huh7 cells with *Pf*CSP@*Pb*CSP sporozoites. Pre-vaccination (circle) and 2 to 3 week post MVA boost (square) boost serum samples were tested at 10% and 20% serum concentrations (left) and the percentage of inhibition observed with 10% serum concentrations was compared to the level of *Pf*CSP specific antibodies measured by ELISA (right) with data analysed with a non-parametric Spearmans test, but no significant correlation was observed. (**d**) Serum samples from human volunteers vaccinated with a ChAd63.*Pf*CSP followed by MVA.*Pf*CSP were tested for the ability to inhibit the invasion of Huh7 cells with *Pf*CSP@*Pb*UIS4 sporozoites. Pre-vaccination (circle) and 2 to 3 week post MVA boost (square) boost serum samples were tested at 2% and 10% serum concentrations (left) and the percentage of inhibition observed with 10% serum concentrations was compared to the level of *Pf*CSP specific antibodies measured by ELISA (right) with data analysed with a non-parametric Spearmens test, but no significant correlation was observed.

To determine whether the differences in inhibition observed with the different parasites strains was related to antibody titre, serum samples showing a range of antibody titres, due to different recombinant vaccine (viral vector vs VLP) or viral vectored vaccine dose, were then tested against both parasite lines (although often in separate experiments due to parasite availability). Consistent with previous experiments, inhibition was observed against both the *Pf*CSP@*Pb*CSP and *Pf*CSP@*Pb*UIS4 parasites with serum from mice vaccinated with a simian adenovirus serotype 63 (ChAd63) expressing CSP followed at least 6 weeks later by a boost vaccination of modified vaccinia Ankara (MVA) expressing *Pf*CSP (ChAd63-MVA *Pf*CSP) (Fig. 4a left, Fig. 4b left). However when mice were vaccinated with a VLP expressing *Pf*CSP, known as R21, which induces high titres of anti-*Pf*CSP antibodies^25^, no inhibition of invasion of *Pf*CSP@*Pb*UIS4 parasites was observed (Fig. 4b left, Figure S3a). In contrast a high level of inhibition of the replacement *Pf*CSP@*Pb*CSP parasites was observed with the same serum samples, even at low serum dilutions (Fig. 4a left) and a positive correlation was observed between antibody titre and inhibition when replacement *Pf*CSP@*Pb*CSP parasites were used in the assay (Fig. 4a right). In contrast, a negative correlation between percentage inhibition and antibody titre was observed when the addition parasites *Pf*CSP@*Pb*UIS4 were used in the assay (Fig. 4b right). The effect was not experiment dependant, as a significant correlation was observed when only a small number of samples were tested together in a single experiment (Figure S3a right). Importantly the inhibition observed in this assay was shown to be antigen specific, as serum samples simultaneously tested against *Pf*CSP@*Pb*CSP, *Pf*CSP@*Pb*UIS4 or GFP parasites, inhibition was only observed against *Pf*CSP@*Pb*CSP parasites (Figure S3b). In addition, serum from mice vaccinated with a different *P*. *falciparum* antigen (UIS3) did not show any inhibition against *Pf*CSP@*Pb*CSP or *Pf*CSP@*Pb*UIS4 parasites (Figure S3c).

Serum samples from human volunteers vaccinated with ChAd63-MVA expressing *Pf*CSP were also assessed for their ability to inhibit invasion of both *Pf*CSP expressing parasites into hepatocyte cell lines. When serum samples were tested for inhibition of the replacement parasite *Pf*CSP@*Pb*CSP, a significant increase post-vaccination was only observed with the 10% serum dilutions (Fig. 4c left) and a positive trend (albeit not statistically significant) towards an increase in inhibition with higher anti-*Pf*CSP antibodies was also observed (Fig. 4c right). When serum was tested for inhibition of the additional copy parasites *Pb*CSP@*Pb*UIS4, an increase in the percentage of inhibition was observed between the pre-vaccination and post-vaccination samples, but was only statistically significant at the 10% serum dilutions (Fig. 4d left), and a significant correlation between the level of anti-*Pf*CSP antibodies and inhibition was also observed (Fig. 4d right).

### The ISI assay using antibodies induced by vaccination with viral vectors expressing *Pf*TRAP

Having observed *Pf*CSP specific inhibition of *Pf*CSP expressing sporozoites with serum from viral-vector vaccinated individuals, we wished to test for the ability to inhibit sporozoite invasion of *P*. *berghei* sporozoites expressing other *P*. *falciparum* antigens. Although CD8^+^ T cells have been shown to play the major role in TRAP specific protection in both mice and humans^26–29^, vaccination with ChAd63-MVA has been shown to induce anti-*Pf*TRAP antibodies in mice, macaques and humans^4,30^, in addition to high frequencies of CD8^+^ T cells. To determine whether inhibition could be observed across species in the ISI assay with an additional *P*. *falciparum* antigen, we tested serum samples from ChAd63-MVA *Pf*TRAP vaccinated mice, macaque and humans, for the ability to inhibit *Pf*TRAP expressing *P*. *berghei* chimeric parasites. Given inhibition against *Pf*CSP@*Pb*UIS4 was observed with samples from ChAd63-MVA vaccinated mice and binding of serum from ChAd63-MVA ChAd63 *Pf*TRAP vaccinated mice to the surface of chimeric parasites has been shown^31^, we were confident that *Pf*TRAP expression on the parasites would be high enough to observe inhibition in this assay.

**Figure 5:**
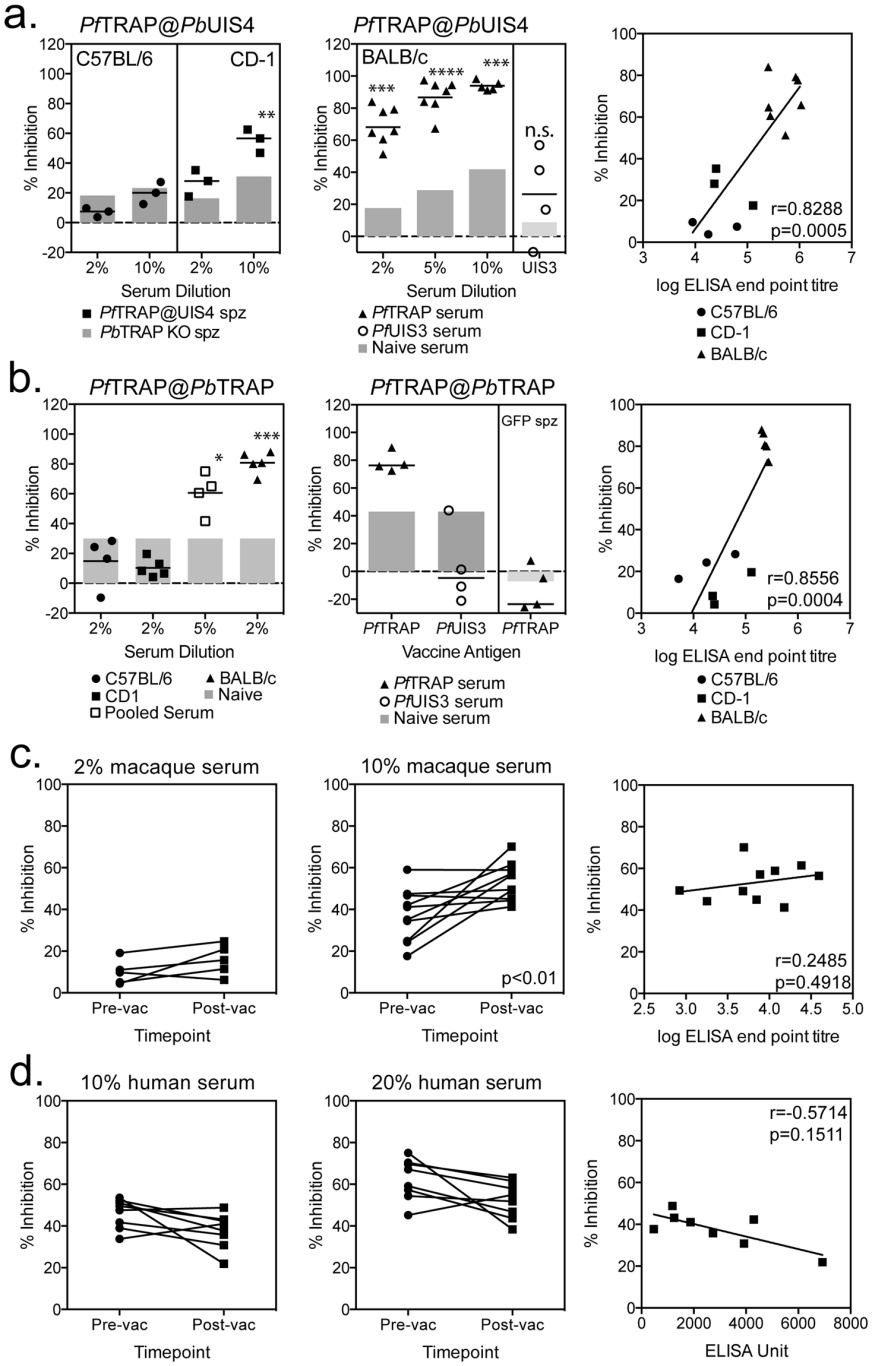
Inhibition of *PbPf*TRAP sporozoite invasion with serum from *Pf*TRAP vaccinated mice, macaques and humans. (**a**) C57BL/6, CD-1 or BALB/c mice immunised with 10^8^ iu ChAd63-*Pf*TRAP and boosted with 10^6^ pfu MVA-*Pf*TRAP (C57BL/6 or CD-1) or 10^7^ pfu MVA-*Pf*TRAP (BALB/c) at least 6 weeks later, with serum harvested approximately 2 weeks after the MVA boost. In a separate experiment, BALB/c mice vaccinated with 10^8^ iu ChAd63-*Pf*UIS3 and boosted with 10^7^ pfu MVA-*Pf*UIS3 were used as an irrelevant antigen control (middle). Huh7 cells were infected with *Pf*TRAP@*Pb*UIS4 sporozoites mixed with increasing concentrations of serum prior to harvesting cells to determine *P*. *berghei* infectivity by flow cytometry 24 to 28 hours later. Graphs represent the percentage of inhibition observed with serum samples from C57BL/6 and CD-1 (left) or BALB/c (middle) vaccinated mice, grey bars denote the level of inhibition observed with naïve serum or inhibition observed against *P*. *berghei* that does not express *Pb*TRAP (left). Data in each graph was analysed with a two-way ANOVA and post-hoc Sidaks multiple comparison, asterisk denote the level of significance comparing *Pf*TRAP samples against naïve serum samples, *p < 0.05, ***p < 0.001, ****p < 0.0001. Inhibition of 2% serum samples plotted against the level of *Pf*TRAP specific antibodies measured by endpoint ELISA (right) with data analysed with a Spearmans non-parametric correlation test, a significant positive correlation was observed (p = 0.0005). (**b**) C57BL/6, CD-1 or BALB/c mice immunised with 10^8^ iu ChAd63-*Pf*TRAP and boosted with 10^6^ pfu MVA-*Pf*TRAP (C57BL/6 or CD-1) or 10^7^ pfu MVA-*Pf*TRAP (BALB/c) at least 6 weeks later, with serum harvested approximately 2 weeks after the MVA boost. BALB/c mice vaccinated with 10^8^ iu ChAd63-*Pf*UIS3 and boosted with 10^7^ pfu MVA-*Pf*UIS3 were used as an irrelevant antigen control (middle). Huh7 cells were infected with *Pf*TRAP@*Pb*UIS4 or GFP only (middle) sporozoites and cells harvested between 24 and 28 hours later to determine *P*. *berghei* infectivity by flow cytometry. Graphs represent the percentage of inhibition observed with serum samples from C57BL/6 and CD-1 or BALB/c (left) vaccinated mice, grey bars denote the level of inhibition observed with naïve serum. Inhibition of 2% serum samples plotted against the level of *Pf*TRAP specific antibodies measured by endpoint ELISA (right) with data analysed with a Spearmans non-parametric correlation test, a significant positive correlation was observed (p = 0.0004). Data in each graph was analysed with a two-way ANOVA and post-hoc Sidaks multiple comparison, asterisk denote the level of significance comparing PfTRAP samples against naïve serum samples, *p < 0.05, ***p < 0.001. (**c**) Rhesus macaques were vaccinated with ChAd63.ME-TRAP followed 8 weeks later by an MVA.ME-TRAP boost were tested for the ability to inhibit invasion of Huh7 cells by *Pf*TRAP@*Pb*UIS4 sporozoites in the standard ISI assay. Pre-vaccination (circle) and 3 week post-MVA boost (square) serum samples were tested at 2% (left) and 10% (middle) serum dilutions, with % inhibition of 10% serum samples (right) compared to the level of post-vaccination TRAP specific antibodies measured by ELISA (right). Data was analysed with a non-parametric Spearmans correlation test, but no significant correlation was observed. (**d**) Serum samples from human volunteers vaccinated with a ChAd63.ME-TRAP followed by MVA.ME-TRAP were tested in the ISI assay for the ability to inhibit the invasion of Huh7 cells with *Pf*TRAP@*Pb*UIS4 sporozoites. Pre-vaccination (circle) and 2 to 3 week post MVA boost (square) boost serum samples were tested at 10% (left) and 20% (middle) serum concentrations and the percentage of inhibition compared to the level of TRAP specific antibodies measured by ELISA (right) with data analysed with a non-parametric Spearmans test, but no significant correlation was observed.

Serum samples were obtained from C57BL/6 and CD-1 mice vaccinated with a heterologous ChAd63-MVA vaccination regimen of vectors expressing the lead liver-stage malaria antigen ME-TRAP, which had previously been shown to induce high titres of anti-*Pf*TRAP antibodies^30^. Although a small increase in the percentage of sporozoite inhibition was observed at 2% serum concentrations, we only observed a significant increase in inhibition, above the level of observed with *P*. *berghei* sporozoites in which *Pb*TRAP had been deleted, with CD1 serum samples at a 10% serum concentration (Fig. 5a left). In a separate experiment, serum samples from ChAd63-MVA *Pf*TRAP vaccinated BALB/c mice were tested at a final concentration of 2, 5 or 10% serum. While the highest level of inhibition was observed with 10% serum, sporozoite inhibition was observed at both lower serum concentrations (Fig. 5a middle). Importantly, serum from mice vaccinated with an alternative *P*. *falciparum* antigen, UIS3, showed no inhibition against *Pf*TRAP@*Pb*UIS4 parasites (Fig. 5a middle). The ability of serum to block sporozoite invasion did not appear to be related to the strain of mice or vaccine insert (ME-TRAP vs TRAP), but the level of antibodies induced by vaccination, as a significant correlation between antibody ELISA titre and percentage inhibition was observed (Fig. 5a right).

Mouse serum samples were also tested against a chimeric *P*. *berghei* line that has the endogenous *trap* gene replaced with the *P*. *falciparum trap* gene (called *Pf*TRAP@*Pb*TRAP). Sporozoites of this line only express *P*. *falciparum* TRAP (*Pf*TRAP) and it produces normal number of sporozoites and infectivity in mice (Salman *et al*., manuscript in preparation). Consistent with *Pf*TRAP@*Pb*UIS4 parasites, no significant inhibition above background (naïve BALB/c serum) with 2% serum from C57BL/6 or CD1 mice was observed, while pooled CD1 serum tested at 5% dilution and 2% serum from BALB/c mice showed significant inhibition above background (Fig. 5b left). Importantly the inhibition against *Pf*TRAP@*Pb*TRAP was shown to be antigen specific, as mice vaccinated with an alternative *P*. *falciparum* antigen, UIS3, did not show inhibition above background, nor did *Pf*TRAP vaccinated serum samples show inhibition against *P*. *berghei* sporozoites expressing only GFP (Fig. 5b middle). In addition, the percentage of inhibition observed in this assay was shown to correlate with *Pf*TRAP antibodies levels (Fig. 5b right).

Serum samples from ChAd63-MVA ME-TRAP vaccinated rhesus macaques^30^ and human volunteers^32^ were also tested for their ability to inhibit *Pf*TRAP@*Pb*UIS4 sporozoites. However, high levels of inhibition observed with the pre-vaccination samples made it difficult to observe a clear effect of vaccination with either the macaque (Fig. 5c) or human (Fig. 5d) serum samples. A small but significant difference between pre and post-vaccination serum samples was observed for macaque samples at 10% dilution (Fig. 5c middle), but not 2% serum dilution (Fig. 5c left), and there was no significant correlation between *Pf*TRAP antibodies levels and the percentage of inhibition (Fig. 5c right). Due to the low-level inhibition and high background observed with serum from macaques and humans, we chose not to test samples against *Pf*TRAP@*Pb*TRAP sporozoites. Given the low titres of antibodies observed against *Pf*TRAP, it would suggest antibody titres need to be above a threshold to observe an antigen specific effect in the ISI assay.

## Discussion

A versatile, robust and relative-high throughput *in vitro* functional assessment of antibody-mediated inhibition of *P*. *falciparum* sporozoite invasion of hepatocytes is still lacking. In this study we have made use of recent advances in transgenic *P*. *berghei* technology to develop a robust *in vitro* inhibition of sporozoite invasion (ISI) assay for the functional assessment of antibodies against *P*. *falciparum* antigens. By utilising fluorescent/luminescent *P*. *berghei* parasite that express *P*. *falciparum* antigens, we can circumvent the challenges conducting experiments with *P*. *falciparum* sporozoites, such as the paucity of cell lines that are permissible infection and the lack of suitable reporter *P*. *falciparum* lines that can be used to rapidly quantify parasite development in infected hepatocytes (i.e. by flow cytometry).

Having identified the optimal cell lines, culture conditions and flow acquisition time-points, we employed the ISI assay to test the inhibitory effect of antibodies against *P*. *falciparum* CSP using chimeric *P*. *berghei* parasites expressing *P*. *falciparum* CSP. CSP is the most abundant antigen expressed on the surface of sporozoites and the basis of the RTS,S vaccine. RTS,S is a VLP based vaccine that consists of a repeat and T-cell epitope containing regions of *Pf*CSP fused to hepatitis B surface antigen and protection after vaccination with RTS,S in combination with the adjuvant AS01 has been shown to correlate with vaccine induced anti-*Pf*CSP antibody levels^9^. In this study, we used two chimeric parasites; one in which the endogenous *csp* gene has been replaced with the *P*. *falciparum csp* gene (’replacement’ chimeric parasites). The other line has the *P*. *falciparum csp* gene expressed under the control of liver-stage promoter UIS4 and therefore in this line both *P*. *berghei* and *P*. *falciparum* CSP are expressed in sporozoites. Such ‘additional copy’ chimeric parasites expressing *P*. *falciparum* proteins have been used to analyse protective immunity, *in vivo*, in mice that have been immunized with vaccines directed against the corresponding *P*. *falciparum* antigens^31^. Specifically, in the case where a *P*. *falciparum* antigen does not have a *P*. *berghei* homolog, the only option for generation of a chimeric *P*. *berghei* is to express the *P*. *falciparum* antigen as an additional copy^31^.

Preliminary experiments, comparing protection after challenge of immunized mice with 3 different antigens with either ‘additional copy’ or ‘replacement’ chimeric parasites revealed the same levels of protection (Salman, unpublished results). In our ISI assay we observed differences in inhibition of invasion of hepatocytes using sporozoites of either additional copy or replacement parasites. Using serum from mice and humans vaccinated with ChAd63-MVA vectors expressing *Pf*CSP, we observed inhibition of sporozoite invasion with both the additional copy and replacement chimeric sporozoites. Interestingly, when using the additional copy chimeric sporozoites, an inverse relationship between the level of anti-*Pf*CSP antibodies and sporozoite invasion was observed. CSP is a critical protein, which once proteolytically cleaved is involved in essential steps involved in host-cell invasion. In the presence of cleavage inhibitors sporozoites are unable to invade hepatocytes^33^. The inverse correlation between antibody titre and invasion observed with the additional copy chimeric sporozoites suggests that in conditions of high anti-*Pf*CSP antibodies, where blocking of sporozoite invasion would be mediated by blocking *Pf*CSP protein cleavage, parasites can use *Pb*CSP-mediated invasion of hepatocytes. This increasing lack of inhibition observed with additional copy chimeric sporozoites was observed with both the monoclonal antibody against *P*. *falciparum* CSP (Fig. 4a) and with serum samples from mice vaccinated with a *Pf*CSP-expressing VLP, which induces the highest levels of anti-*Pf*CSP antibodies. In contrast, a significant positive correlation between antibody titre and the level of sporozoite invasion was observed with the replacement sporozoites which only express *Pf*CSP (Fig. 4a). A possible explanation why protection is observed *in vivo* after challenge of immunized mice with sporozoites of the additional copy chimeric parasites^25^ is that *in vivo* protection can be dependent on multiple factors, such as neutralising sporozoites, opsonisation or reducing growth within the hepatocyte^34^. It is important to note that since *Pb*UIS4 drives *P*. *falciparum* antigen expression in the additional copy parasites and it is one of the most abundant mRNA transcripts present in salivary gland sporozoites^35–37^, it is likely that the level of *P*. *falciparum* antigen expression in addition parasites will be higher than the *P*. *berghei* homolog or observed with the natural promoter in *P*. *falciparum* sporozoites. It is therefore possible that performing the ISI assay with addition parasites may show greater levels of inhibition that would be observed with replacement *P*. *berghei* chimeric or *P*. *falciparum* sporozoites. Given the inverse relationship between antibody titre and inhibition of invasion observed with additional copy vs replacement chimeric *Pf*CSP sporozoites, in addition to the likelihood that there will be overexpression of the *P*. *falciparum* antigen in addition parasites, it will be essential, where possible, to use replacement chimeric sporozoites in future ISI assays, particularly where the vaccination regimen is capable of inducing high titre antibodies.

Humanised mice, specifically those with livers engrafted with human hepatocytes, have been used to analyse inhibition of *P*. *falciparum* sporozoite invasion of hepatocytes, both using anti-*Pf*CSP monoclonal antibodies^38^ and with sera from human volunteers immunized after infection with sporozoites, by mosquito bites, and treated with chloroquine (DAP)^39^. However these experiments are expensive and laborious, primarily due to the purchase cost of the humanised mice or time and staffing required to maintain and engraft humanised mice in house, and this very much limits their use in screening large numbers of serum samples. With the ability to generate chimeric *P*. *berghei* sporozoites that express *P*. *falciparum* ^31^ and *P*. *vivax* ^40^ antigens as either replacement or additional copies^17^, the ISI assay as described here can be used to rapidly test serum from human clinical vaccine trials and other pre-clinical vaccine studies where humans and animals have been vaccinated with different human malaria vaccines.

Although viral-vectored vaccines have been used primarily for the induction of high levels of CD8^+^ T cells against the liver-stage of malaria, heterologous prime-boost vaccination with ChAd63 followed by MVA also induces strong antibodies responses^41^. Therefore we tested serum samples from mice, rhesus macaques and humans that had been vaccinated with these viral vectors expressing either *Pf*CSP or *Pf*TRAP to analyse inhibition of invasion of *P*. *berghei* sporozoites expressing either *Pf*CSP or *Pf*TRAP. Serum samples from mice vaccinated with either ChAd63-MVA *Pf*CSP or *Pf*TRAP both displayed inhibition against the corresponding chimeric *P*. *berghei* parasites, and inhibition correlated with the level of anti-*Pf*CSP or anti-*Pf*TRAP antibodies. However the level of antibodies induced in macaques and humans with ChAd63-MVA *Pf*TRAP vaccination was too low to observe an inhibitory effect on sporozoite invasion and only a small inhibitory effect was observed when using human serum after ChAd63-MVA *Pf*CSP vaccination. Due to the low-level of inhibition observed with *Pf*TRAP@*Pb*UIS4 parasites, where *Pf*TRAP would likely be over-expressed and potentially give higher levels of inhibition, we chose not to test these same samples with *Pf*TRAP@*Pb*TRAP when they became available. Antibodies induced by immunisation with ChAd63-MVA expressing *Pf*CSP are known to be lower than induced by vaccination with RTS,S^32^, and this would most likely explain why we observed significant increase in ISI inhibition when we used serum samples from mice vaccinated with the other, more recently described, *Pf*CSP VLP vaccine, R21^25^. Therefore, it will be of great interest to use the ISI assay to directly compare the inhibitory capacity of serum collected from either RTS,S or R21 vaccinated individuals in future clinical trials.

The low level inhibition observed with low titre serum samples (macaques, human and *Pf*TRAP vaccinated C57BL/6 and CD1 mice) highlights the need to include pre-vaccination or naïve serum controls in each test to ensure inhibition is specific. This is particularly true as the higher the quantity of serum in the assay, the greater the non-specific effect of serum. In addition, varying levels of inhibition seen with naïve serum in different experiments may suggest serum quality could affect the assay readout, although this has not formally been tested in this study. Despite the background inhibition observed in this assay, the data clearly shows that when strong inhibition is observed, the inhibition is antigen specific, as we do not observe inhibition with irrelevant antigens, nor was inhibition against wild-type *P*. *berghei* parasites observed with *Pf*TRAP and *Pf*CSP serum samples. This however will not always be the case, particularly when *P*. *falciparum* antigens have reported cross species reactivity, for example *P*. *falciparum* CelTOS^42^. In these instances it will be important to use replacement parasites to limit the cross species effect.

In this study we have developed a robust *in vitro* ISI assay by making use of the advances in transgenic *P*. *berghei* parasite technology, that can be used to functionally assess the invasion inhibitory capacity of antibodies against *P*. *falciparum* antigens. Until such time that brightly fluorescent *P*. *falciparum* sporozoites and, importantly, a permissive (preferably immortalised) human hepatocyte cell line becomes readily available, this *in vitro* ISI assay should prove to be a valuable addition to the toolkit of assays to functionally assess vaccine induced antibodies against *P*. *falciparum* antigens.

## Materials and Methods

### Ethics statement

All animal work was conducted in accordance with the UK Animals (Scientific Procedures) Act 1986 and approved by the University of Oxford Animal Care and Ethical Review Committee for use under Project License 30/2889 and P9804B4F1. Animals were group housed in individually ventilated cages under specific pathogen free conditions, with constant temperature, humidity and with a 12:12 light-dark cycle (8am to 8 pm). For induction of short-term anaesthesia, animals were either injected intramuscularly (i.m.) with xylazine and ketamine or anaesthetized using vaporized IsoFlo®. All animals were humanely sacrificed at the end of each experiment by an approved Schedule 1 method. All efforts were made to minimize suffering.

Rhesus macaque serum samples were obtained from an immunogenicity study measuring the improved immunogenicity of ChAd63-MVA ME-TRAP by fusion to human MHC Class II invariant chain^30^. Ethical approval was granted by the University of Wisconsin-Madison IACUC (termed Animal Care and Use Committee) and granted protocol number g00677.

Human serum samples used in this study were obtained from a Phase IIa efficacy study in which individuals were vaccinated with ChAd63-MVA expressing ME-TRAP or *Pf*CSP prior to controlled human malaria challenge (CHMI) with *P*. *falciparum* infected mosquitoes^32^. The study was conducted at the Centre for Clinical Vaccinology and Tropical Medicine, University of Oxford (Oxford, United Kingdom), and at the National Institute for Health Research (NIHR) Wellcome Trust Clinical Research Facility, part of the University of Southampton and University Hospital Southampton National Health Service (NHS) Foundation Trust (Southampton, United Kingdom). Healthy, malaria-naive men and non-pregnant women aged 18–45 years were invited to participate in the study. All volunteers gave written informed consent prior to participation, and the study was conducted according to the principles of the Declaration of Helsinki and in accordance with good clinical practice. All necessary approvals for the study were granted by the United Kingdom National Research Ethics Service, Committee South Central–Oxford A (reference 12/SC/0037), and the United Kingdom Medicines and Healthcare Products Regulatory Agency (reference 21584/0293/001-0001). The trial was registered with Clinical-Trials.gov (referenceNCT01623557).

### Vaccines and Vaccinations

Female BALB/cOlaHsd (BALB/c), C57BL/6JOlaHsd (C57BL/6) or ICR (CD1) mice 6 weeks of age or older (Envigo, UK) were immunized intramuscularly with chimpanzee adenovirus 63 (ChAd63) expressing the relevant antigen, followed at least 6 weeks later with an intramuscular injection of modified Ankara Virus (MVA) expressing the relevant antigen. All vaccines were produced and titred as previously described^43^. To induce high titre antibodies against *P*. *falciparum* CSP, BALB/c mice received 2 doses of 3 μg of a virus-like particle expressing the repeat region and C-terminal domain of *Pf*CSP (R21)^25^ mixed with 12 μg of adjuvant Matrix M 4 weeks apart with serum harvested 2 weeks after the final vaccination. Antibody responses against either *Pf*TRAP or *Pf*CSP were measured by ELISA, with plates coated with *Pf*TRAP protein or a synthetic peptide (Eurogentec) based on the repeat region of the *Pf*CSP with the amino acid sequence *Pf*CSP(NANP)6C^32^.

3D11 (anti-*Pb*CSP monoclonal antibody) and 2A10 (anti-*Pf*CSP monoclonal antibody) hybridomas, obtained from MR4 or BEI resources, were grown in house and supernatant purified over a protein A column following standard technique. Only a Fab preparation of 3D11 was available at the time of optimisation experiments, however it gave comparable results to full length 3D11 mAb when the two preparations were later directly compared (data not shown).

### Hepatocyte Cell Lines

HepG2 (ATCC), Huh7 and HC04 (ATCC) hepatocyte cell lines were propagated in DMEM (Dulbecco’s Modified Eagle’s Medium) or RPMI-1640 supplemented with 10% heat inactivated FCS, 100 U/ml penicillin, 100 μg/ml streptomycin and 2 mM L-glutamine (all reagents obtained from Sigma Aldrich). Cells were cultured at 37 ^o^C and 5% CO_2_ with the addition of TrypLE Express Enzyme (Life Technologies) to aid in detachment of cells from culture plates or flasks. Typically 30000 cells were seeded on a 96 well-flat bottom plate at least 6 hours prior to sporozoite addition.

### Parasite Preparation

*Plasmodium berghei* sporozoites expressing GFP under the control of the EF1a promoter (*Pb*ANKA-GFP_Pbeef1a_, line 507cl1, called *Pb*GFP)^44^, GFP-luciferase (*Pb*ANKA-GFP::Luc_Pbeef1a_230p_, line 507cl1, called *Pb*GFP-Luc), GFP-luciferase and *Pf*CSP under the control of the *P*. *berghei* CSP promoter (*Pb*ANKA-*Pf*CSP(r)_*Pb*CSP_; line 2257cl2, called *Pf*CSP@*Pb*CSP), *Pf*TRAP under the control of the *P*. *berghei* TRAP promoter (*Pb*ANKA-*Pf*TRAP(r)_*Pb*TRAP_; line 2632 cl1, called *Pf*TRAP@*Pb*TRAP) (Salman in preparation) or GFP-luciferase and *P*. *falciparum* antigens under the control of the UIS4 promoter^31^ (*Pf*CSP_*Pb*uis4 GFP::LucPbeef1a_230p_, line 2243cl3, called *Pf*CSP@*Pb*UIS4; *Pf*TRAP_Pbuis4 GFP::LucPbeef1a_230p_, line 2281cl1, called *Pf*TRAP@*Pb*UIS4) were isolated from female *Anopheles stephensi* mosquitoes around 21 days after feeding on *P*. *berghei* blood stage infected donor mouse. To improve sporozoite yields, mosquitoes were fed on a naïve mouse 7 days after the first feed. Salivary glands from infected mosquitoes were manually dissected and kept on ice, with homogenisation and counting sporozoites under phase contrast microscopy performed just prior to plating to maintain sporozoite infectivity. Sporozoites (typically 15000 per well) were mixed on ice with serum samples or monoclonal antibody diluted in medium. For addition of sporozoite to hepatocyte cell lines, culture medium was removed and replaced with mAb/serum and sporozoite mixture (final volume 100μl), prior to centrifugation of the plates at 500 g for 5 minutes to enhance sporozoite entry into hepatocytes^18^ and incubation at 37 ^o^C.

### Assessment of infectivity by flow cytometry

Cells were harvested after overnight incubation when using *Pb*GFP, or at least 24 hours after addition of sporozoites when using *Pb*GFP-Luc sporozoites. Typically culture medium was removed from each well and 30 µL of trypsin (TrypLE Express Enzyme, Life Technologies) added and incubated for 10–15 minutes, prior to resuspension in 1%BSA (10% Fetal Calf Serum) in PBS and transferred to a FACS tube or to a 96-well U bottom plate for acquisition. 4′,6-diamidino-2-phenylindole dihydrochloride (DAPI, final concentration 1 μg/ml, Sigma Aldrich) was added just prior to acquisition for the exclusion of dead cells. Samples were acquired with a LSR II^TM^ flow cytometer (BD Biosciences) using FACSDIVA^TM^ software V 6.2 (BD Biosciences). *P*. *berghei* infected cells were identified by gating on viability and size, removing doublets and gating on GFP positive but PE (autofluorescene) negative cells (Figure S3).

### Statistical analysis

Cytometer acquisitions files (.fcs) were analysed with FlowJo.V 9.7.6 (Tree Star). The percentage of sporozoite inhibition was calculated as a reduction in the percentage of infected cells observed in untreated wells (average) compared to the percentage of infected cells observed in the presence of serum/mAb.

Graphs and statistical analysis was performed using GraphPad Prism 6.0. A potential correlation between two parameters was evaluated using a nonparametric Spearman correlation. When comparing the percentage inhibition between groups, Kruskal-Wallis test with Dunn’s multiple comparisons test were used. To evaluate whether there was an effect of vaccination in macaque and human samples, blocking values pre- and post-vaccination (paired data) were analysed with a Wilcoxon matched-pairs signed rank test (two tailed). P-values lower than 0.05 were considered to be significant. To determine if there was a specific effect of mouse serum compared to naïve controls at different serum dilutions or from different vaccination regimens, data was analysed with a two-way ANOVA and post-hoc Sidaks multiple comparison test.

## Electronic supplementary material

Supplementary Data
