# Supplementary material for: An in vitro assay to measure antibody-mediated inhibition of P. berghei sporozoite invasion against P. falciparum antigens
Source: Sci Rep. 2017 Dec 5;7:17011. doi: 10.1038/s41598-017-17274-5 (PMC5717233; doi:10.1038/s41598-017-17274-5)
Supplement: Supplementary file 1 — Supplementary Data [file 41598_2017_17274_MOESM1_ESM.pdf]

An *in vitro* assay to measure antibody-mediated inhibition of *P. berghei* sporozoite invasion against *P. falciparum* antigens

Ana Rodríguez-Galán[1,2], Ahmed M Salman[1], Georgina Bowyer[1], Katharine A. Collins[1], Rhea J Longley[1,3], Florian Brod[1], Marta Ulaszewska[1], Katie J Ewer[1], Chris J Janse[4], Shahid M Khan[4], Julius C Hafalla[2], Adrian VS Hill[1], Alexandra J Spencer[1*]

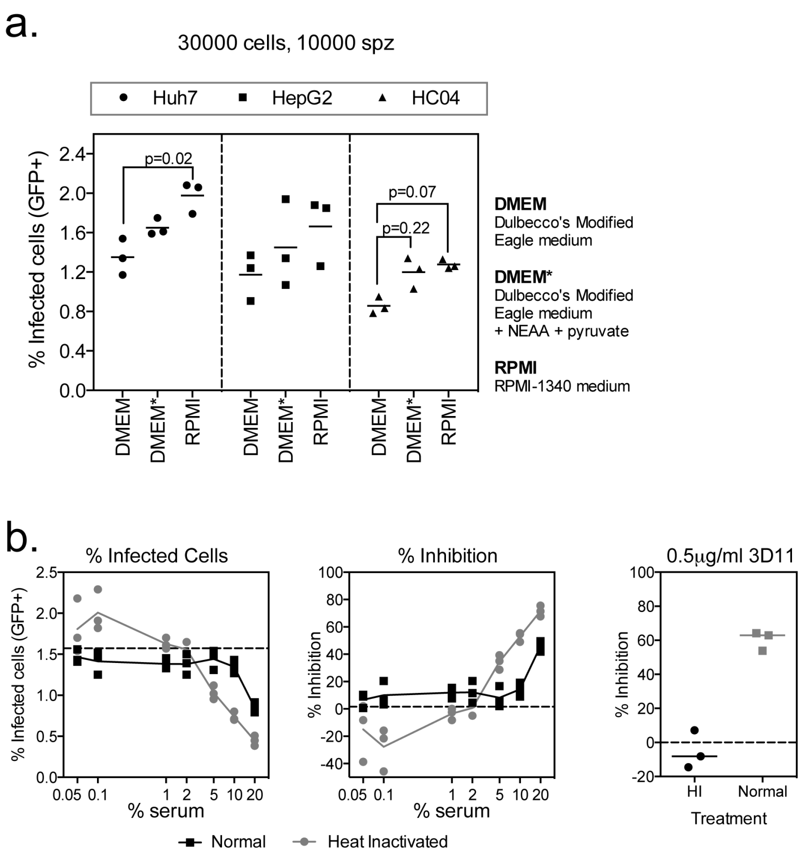

Figure S1: Optimising culture conditions and treatment of serum for the ISI assay.

a.) 30000 cells (Huh7, HepG2 or HC04) were seeded per well and infected with 10000 *Pb*GFP sporozoites and cultured in DMEM, DMEM* or RPMI medium overnight prior to harvesting and analysis of the percentage of infected cells by flow cytometry. Data was analysed with Kruskal-Wallis test, Dunn's multiple comparisons test (p-values below 0.3 are included in the graph).

b.) 15000 sporozoites (*Pb*GFP-Luc) were added to 30000 Huh7 cells in the presence of various concentrations (0-20%) of serum from naive BALB/c mice or 1μg/mL mAb-*Pb*CSP that had either been heat inactivated (HI) (black) or untreated (grey). Graphs represent the

percentage of infected cells (left) or percentage of inhibition observed with increasing concentrations of naïve BALB/c serum (middle) or 1μg/ml mAb-*Pb*CSP 3D11 (right).

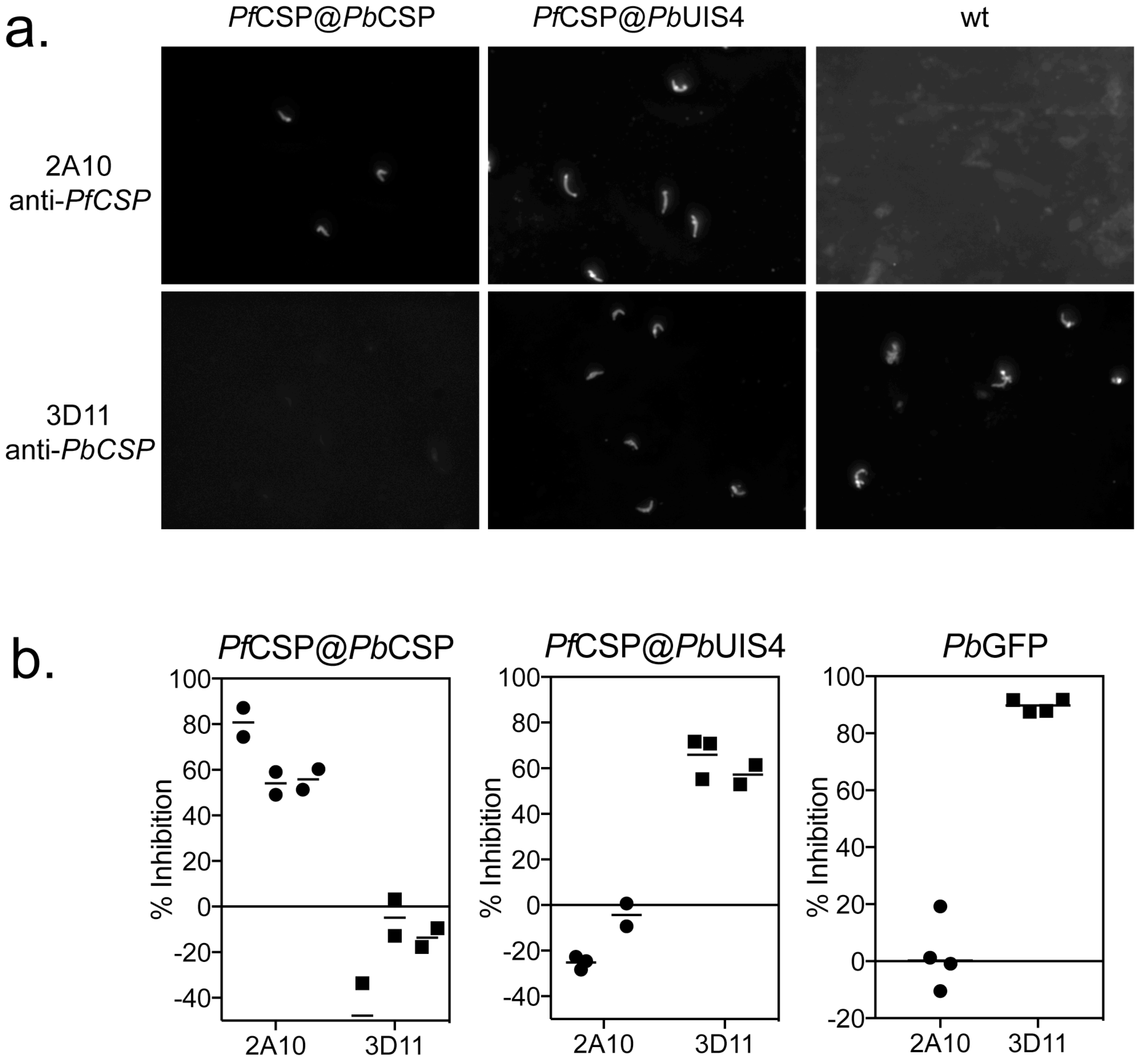

Figure S2: Specificity of 2A10 and 3D11 monoclonal antibodies

a.) *Pf*CSP@*Pb*CSP, *Pf*CSP@*Pb*UIS4 or wild-type *P.berghei* salivary gland dissected sporozoites were stained with 0.1μg/ml of 2A10 or 3D11 monoclonal, followed by detection of bound antibody with anti-mouse Alexa488.

b.) Graphs represent the percentage of inhibition against *Pf*CSP@*Pb*CSP, *Pf*CSP@*Pb*UIS4 or GFP sporozoites observed across different experiments following the addition of either 2A10 or 3D11 monoclonal antibody. GFP parasite data was acquired in two separate experiments.

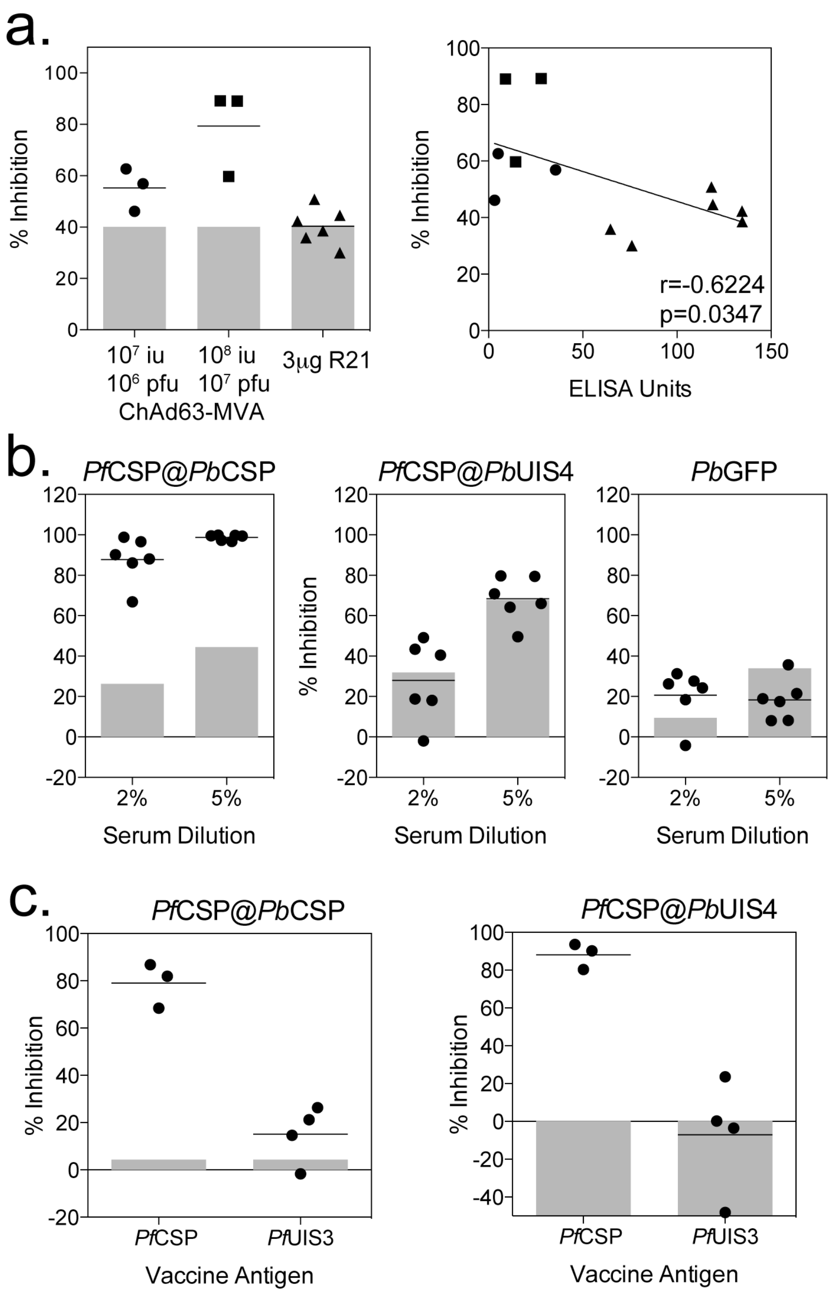

Figure S3: Specificity of sporozoite inhibition observed by anti-*Pf*CSP serum samples

a.) 30000 Huh7 cells were seeded per well and rested overnight prior to the addition of 15000 *Pf*CSP@*Pb*UIS4 sporozoite together with 2% serum from BALB/c mice vaccinated with 10^7 iu ChAd63.CSP 10^6 MVA.CSP (filled circle), 10^8 iu ChAd63.CSP 10^7 pfu MVA.CSP (closed squares), 3μg R21 in Matrix M or naïve mice (grey bars). The percentage of inhibition was compared to antibody titres measured by ELISA (right) and analysed with a two-tailed Spearmans correlation.

b.) In a single experiment, 30000 Huh7 cells per well were seeded overnight prior to the addition of 2% or 5% serum dilutions and 15000 *Pf*CSP@*Pb*CSP, *Pf*CSP@*Pb*UIS4 or *Pb*GFP

sporozoites. Cells were harvested at least 24 hours later to measure the frequency of GFP⁺ cells by flow cytometry and to calculate the percentage of inhibition of the samples against the three different *P. berghei* lines. The data from *Pf*CSP@*Pb*UIS4 and *Pf*CSP@*Pb*CSP is also presented in Figure 4 of this manuscript. Grey bars represent the inhibition of naïve serum controls.

c.) In a single experiment, 30000 Huh7 cells per well were seeded overnight prior to the addition of 2% dilutions of serum from mice vaccinated with 10^8iu ChA63 followed by 10^7pfu MVA expressing either *Pf*CSP or *Pf*UIS3, and with 15000 *Pf*CSP@*Pb*CSP or *Pf*CSP@*Pb*UIS4 sporozoites. Cells were harvested 24 to 28 hours later to measure the frequency of GFP^+ cells by flow cytometry and to calculate the percentage of inhibition of the samples against the two different *P. berghei* lines. Grey bars represent the inhibition of naïve serum controls.

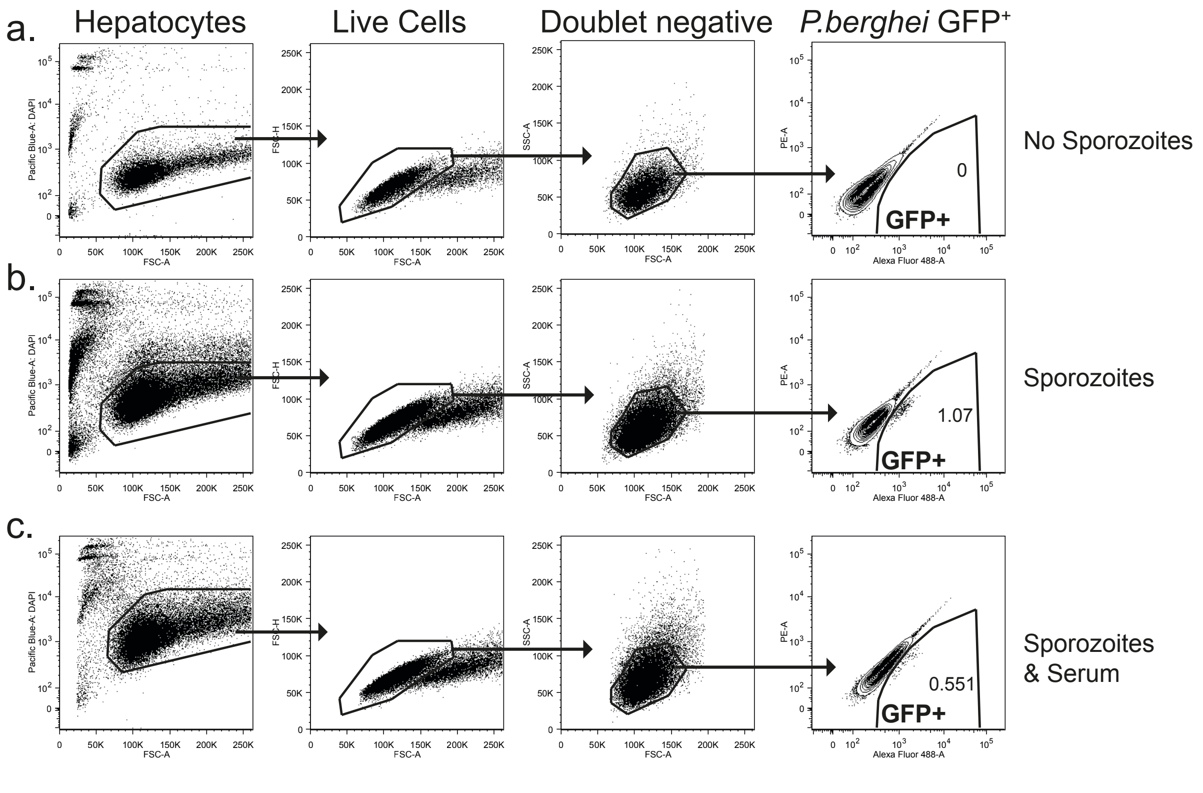

Figure S4: Gating strategy for detection of *P. berghei* infected cells

*Pb*GFP infected hepatocytes were identified by gating for live cells (DAPI negative), size (FSC-A vs SSC-A), removing doublets (FSC-A vs FSC-H) and then gating for GFP^{+} relative to auto-fluoresce in the adjacent PE channel.
